# Association between Platelet-Specific Collagen Receptor Glycoprotein 6 Gene Variants, Selected Biomarkers, and Recurrent Pregnancy Loss in Korean Women

**DOI:** 10.3390/genes11080862

**Published:** 2020-07-29

**Authors:** Hui Jeong An, Eun Hee Ahn, Jung Oh Kim, Chang Soo Ryu, Han Sung Park, Sung Hwan Cho, Ji Hyang Kim, Woo Sik Lee, Jung Ryeol Lee, Young Ran Kim, Nam Keun Kim

**Affiliations:** 1 Department of Biomedical Science, College of Life Science, CHA University, Seongnam 13488, Korea; tody2209@naver.com (H.J.A.); jokim8505@gmail.com (J.O.K.); regis2040@nate.com (C.S.R.); hahnsung@naver.com (H.S.P.); arana006@naver.com (S.H.C.); 2 Department of Obstetrics and Gynecology, CHA Bundang Medical Center, School of Medicine, CHA University, Seongnam 13488, Korea; bestob@chamc.co.kr (E.H.A.); bin0902@chamc.co.kr (J.H.K.); 3 Fertility Center of CHA Gangnam Medical Center, CHA University, Seoul 061, Korea; wooslee@cha.ac.kr; 4 Department of Obstetrics and Gynecology, Seoul National University Bundang Hospital, Seongnam 13620, Korea; leejrmd@snu.ac.kr

**Keywords:** glycoprotein 6 (*GP6*), recurrent pregnancy loss, polymorphism, platelet, miRNA

## Abstract

This paper investigates whether glycoprotein 6 (*GP6*) gene polymorphisms are a risk factor for recurrent pregnancy loss (RPL) in Korean women. Genotypes were determined by polymerase chain reaction-restriction fragment length polymorphism and real-time polymerase chain reaction amplification. We identified five polymorphisms in the *GP6* gene: rs1654410 T>C, rs1671153 T>G, rs1654419 G>A, rs12610286 A>G, and rs1654431 G>A. *GP6* rs1654410 CC was associated with decreased RPL risk (adjusted odds ratio = 0.292, 95% confidence interval = 0.105–0.815, *p* = 0.019), and recessive genotypes were also significantly associated with decreased RPL risk (adjusted odds ratio = 0.348, 95% confidence interval = 0.128−0.944, *p* = 0.038). *GP6* rs1654419 GA was associated with decreased RPL risk (adjusted odds ratio = 0.607, 95% confidence interval = 0.375-0.982, *p* = 0.042), and dominant genotypes were significantly associated with decreased RPL risk (adjusted odds ratio = 0.563, 95% confidence interval = 0.358−0.885, *p* = 0.013). Altogether, the genotype frequencies of *GP6* rs1654410 T>C and *GP6* rs1654419 G>A were significantly different between RPL patients and control participants. Therefore, although *GP6* polymorphisms may be useful as biomarkers of RPL, additional studies with heterogeneous cohorts are required to better understand the influence of GP6 and assess its performance as a biomarker.

## 1. Introduction

Recurrent pregnancy loss (RPL) is defined as two or more consecutive pregnancy losses [1]. Factors that contribute to the etiology of RPL include advanced maternal age, maternal anatomic anomalies, placental anomalies, chromosome abnormalities, endocrine dysfunction, antiphospholipid syndrome, hereditary thrombophilia, psychological trauma, and environmental factors, such as smoking, excessive alcohol consumption, and stress [2]. Moreover, the likelihood of pregnancy loss is 5% higher for women who suffer a miscarriage during their first pregnancy than for healthy subjects [3]. Although many factors that contribute to the etiology of RPL have been identified, the underlying cause remains unknown in the majority of cases. RPL is associated with immune disorders, blood coagulation, and angiogenesis. These factors are related to hemostasis, which in turn contributes to platelet activation. Collagen is a platelet activator that plays an important role in the vascular endothelium and vascular wall. Platelets have two major receptors for collagen: integrins, which play a role in platelet aggregation, and glycoprotein 6 (*GP6*), which is primarily involved in signal transduction and platelet activation [4].

*GP6* is an immunoglobulin-like collagen receptor that is exclusively expressed in platelets and megakaryocytes and is essential for cell activation in matrix proteins. *GP6* is the product of the *GP6* gene, which is located on chromosome 19 (19q13.4) [5]. Since the identification and analysis of the *GP6* gene in the 1990s, a number of single nucleotide polymorphisms (SNPs) have been identified [6].

*GP6* is essential for integrin activation, the formation of stable attachments, and subsequent signaling and platelet aggregation in both *in vitro* and *in vivo* studies [4]. The GP6 is a receptor for collagen and plays a critical role in collagen-induced platelet aggregation and thrombus formation [7]. In fact, several *GP6* polymorphisms (rs1671153, rs1654419, and rs1613662) are associated with an increased risk of platelet aggregation [8,9]. The precise mechanisms regulating GP6 gene molecular variations and aggregation function are largely unknown; however, some studies have suggested that the genetic polymorphisms in GP6 may influence receptor density and platelet function [10,11]. The aim of the present study was twofold: to evaluate the genetic variability of the GP6 gene in control and patients with RPL, and to carry out association analyses of GP6 with two, three, or four pregnancy losses. Specifically, we investigated the association between RPL and five *GP6* polymorphisms (rs1654410 T>C, rs1671153 T>G, rs1654419 G>A, rs12610286 A>G, and rs1654431 G>A) in a population of Korean women. Sticky platelet syndrome (SPS) is the second most common thrombophilia that causes recurrent spontaneous abortions or fetal loss syndrome [12,13]. It was found that there was a significant association between GP6 gene polymorphism and thromboembolic events in SPS patients [14]. Sokol et al. [9] has found that some polymorphisms of GP6 (including rs1654410, rs1671153, rs1654419, rs12610286, and rs1654431) represent a risk factor in patients with SPS and fetal loss. Considering the critical role of GP6 in platelet coagulant activity, the polymorphisms in GP6 may influence pregnancy loss. We demonstrated that *GP6* rs1654410C>T, GP6 rs1654419 G>A, and GP6 rs1654431 G>A SNPs were associated with RPL in this patient cohort.

## 2. Materials and Methods

### 2.1. Participants

Blood samples were collected from 388 patients with RPL (mean age ± SD, 33.20 ± 4.54 years) and 219 control participants (mean age ± SD, 32.75 ± 3.84 years). The RPL patients were recruited from the Department of Obstetrics and Gynecology or Fertility Center of the CHA Bundang Medical Center in Seongnam between March 1999 and February 2010. The women in the control group were also recruited from the CHA Bundang Medical Center and met the following criteria: pregnant, regular menstrual cycles, a history of at least one naturally conceived pregnancy, no history of pregnancy loss, and karyotype 46, XX. The Institutional Review Board of the CHA Bundang Medical Center approved the study, and all patients gave written informed consent. The IRB number for this study was BD2010-123D. All patients had suffered a minimum of two consecutive spontaneous miscarriages, and blood samples were taken based on human chorionic gonadotropin (hCG) levels prior to 20 weeks gestation. The average gestational age was 7.32 ± 2.05 weeks. Pregnancy loss was diagnosed with human chorionic gonadotropin (hCG) testing, ultrasound, and/or physical examination before 20 weeks of gestational age. None of the participants had a history of smoking or alcohol use. Patients with recurrent pregnancy loss due to anatomic, hormonal, chromosomal, infectious, autoimmune, or thrombotic causes were excluded from the study. All study protocols were abided by the recommendations of the Declaration of Helsinki, and written informed consent was obtained from all study participants.

### 2.2. Genotyping

Genomic DNA was extracted from anticoagulated peripheral blood samples using a G-DEX™ Genomic DNA Extraction Kit for blood (iNtRON Biotechnology, Seongnam, Korea) [15]. Five *GP6* (SNPs) were selected using the human genome SNP database (dbSNP, http://www.ncbi.nlm.nih.gov/snp).

For RFLP analysis of the SNPs, the PCR products for GP6T>C rs1654410, GP6T>G, rs1671153, GP6G>Ars1654419 were digested with the restriction enzymes Mbo II, Hph I, and Cse I, respectively. To confirm the three SNPs and validate the RFLP results, 10–20% of the samples were randomly selected, used for a second round of PCR, and analyzed by DNA sequencing using an automatic ABI3730xL DNA analyzer (Applied Biosystems, Forster City, CA, USA). Samples were genotyped by real-time polymerase chain reaction (PCR) using a qPCR kit with the primers listed in Supplementary Table S1. The conditions for the five polymorphisms (rs1654410 T>C, rs1671153 T>G, rs1654419 G>A, rs12610286 A>G, and rs1654431 G>A) are also listed in Supplementary Table S1.

### 2.3. Assessment of Plasma Plasminogen Activator Inhibitor-1 (PAI-1), Homocysteine, Total Cholesterol, Uric Acid, and Blood Coagulation Status

Plasma PAI-1, homocysteine, total cholesterol, and uric acid were measured in blood collected from RPL patients. Plasma was separated by centrifuging whole blood samples at 1000 × *g* for 15 min. PAI-1 levels were determined using a human serpin E1/PAI-1 immunoassay (R&D Systems, Minneapolis, MN, USA). Uric acid and total cholesterol were measured using commercially available enzymatic colorimetric tests (Roche Diagnostics, GmbH, Mannheim, Germany). Homocysteine was measured using a fluorescence polarization immunoassay using the Abbott IMx analyzer (Abbott Laboratories, Abbott Park, IL, USA).

### 2.4. Statistical Analysis

**Figure 1:**
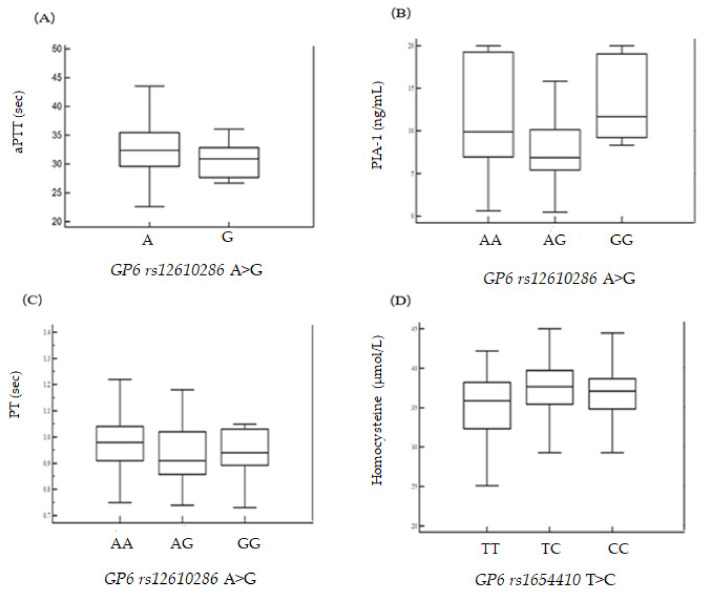
Analysis of variance for aPTT, PAI-1, PT, and homocysteine levels according to *GP6* polymorphisms (PL ≥ 3). (**A**) aPTT levels were significantly different (*p* = 0.047) between *GP6 rs12610286* AA (mean ± SD, 32.36 ± 4.30), AG (32.73 ± 3.99), and GG (30.49 ± 3.02). (**B**) PAI-1 levels were significantly different (*p* = 0.0003) between *GP6 rs12610286* AA (mean ± SD, 11.75 ± 6.01), AG (7.89 ± 4.34), and GG (13.26 ± 4.88). (**C**) PT (sec) levels were significantly different (*p* = 0.045) between *GP6 rs12610286* AA (mean ± SD, 0.97 ± 0.10), AG (0.94 ± 0.12), and GG (0.95 ± 0.09). (**D**) Homocysteine levels were significantly different (*p* = 0.026) between *GP6 rs1654410* TT (mean ± SD, 36.54 ± 3.54), TC (37.12 ± 3.88), and CC (35.04 ± 4.31).

Differences in the frequency of *GP6* polymorphisms between the control and patient groups were assessed using Fisher’s exact test and a logistic regression model. Odds ratios (ORs), adjusted odds ratios (AORs), and 95% confidence intervals (CIs) were used to examine the association between *GP6* polymorphisms and RPL risk. The data are presented as mean ± standard deviation (SD) for continuous variables or as percentages for categorical variables. Statistical analyses were carried out using MedCalc version 12.1.4 (MedCalc Software bvba, Mariakerke, Belgium) or GraphPad Prism 4.0 (GraphPad Software, Inc., San Diego, CA, USA). The HAPSTAT program (v.3.0, www.bios.unc.edu/~lin/hapstat/) was used with a strong synergistic effect to estimate the frequency of polymorphic haplotypes, *p*-values of <0.05 were considered statistically significant. The false discovery rate (FDR) was also used to adjust for multiple comparisons; associations with an FDR-corrected *p*-value of <0.05 were considered statistically significant [16]. Genetic interaction analysis was performed with the open-source multifactor dimensionality reduction (MDR) software package (v.2.0) available from www.epistasis.org. The MDR method consists of two main steps [17,18,19]. During ANOVA analysis, the Kruskal–Wallis test was used for small sample sizes and/or when the P-value of Levene’s test was less than 0.05 (Figure 1).

## 3. Results

The clinical profiles and demographic characteristics of RPL patients and control participants are presented in Table 1. The mean ages of RPL and control patients were 32.75 ± 3.84 and 33.20 ± 4.54 years, respectively. When comparing the RPL patients with the control participants, the RPL patients had significantly higher platelet counts (PLT), prothrombin times (PT), activated partial thromboplastin times (aPTT), luteinizing hormone (LH) levels (mIU/mL, mean ±SD), and estradiol (E2) levels (pg/mL, mean ±SD) (all *p* < 0.05; Table 1). However, many of the factors measured here undergo changes during the course of pregnancy.

The genotype frequencies and ORs of the *GP6* polymorphisms rs1654410 T>C, rs1671153 T>G, rs1654419 G>A, rs12610286 A>G, and rs1654431 G>A in control and RPL patients are listed in Table 2. All of the genotype frequencies that were analyzed showed polymorphisms and occurred in Hardy–Weinberg equilibrium (HWE) in both groups. The map of the GP6 gene is shown in Figure S1. We found a significant association between *GP6* polymorphisms and three or more RPLs: *GP6* rs1654410 T>C (TT versus CC: AOR = 0.292; 95% CI = 0.105–0.815; *p* = 0.019), (TT + TC versus CC: AOR = 0.348; 95% CI = 0.128–0.944; *p* = 0.038). *GP6* rs1654419 G>A (GG versus GA: AOR = 0.607; 95% CI = 0.375–0.982; *p* = 0.042), (GG versus GA + AA: AOR = 0.563; 95% CI = 0.358–0.885; *p* = 0.013), and *GP6* rs1654431 G>A (GG versus GA: AOR = 1.663; 95% CI = 1.007–2.746; *p* = 0.047), (GG versus GA + AA: AOR = 1.746; 95% CI = 1.082–2.818; *p* = 0.022) compared with the control group.

When the number of RPLs was increased to four or more, only two *GP6* variants remained associated with RPL risk: *GP6* rs1654419 G>A (GG versus GA: AOR = 0.506; 95% CI = 0.273–0.940; *p* = 0.031), (GG versus GA + AA: AOR = 0.473; 95% CI = 0.265–0.846; *p* = 0.012) and *GP6* rs1654431 G>A (GG versus GA: AOR = 1.967; 95% CI = 1.052–3.677; *p* = 0.034), (GG versus GA + AA: AOR = 1.856; 95% CI = 1.013–3.400; *p* = 0.045). However, after FDR-p correction, the association was not statistically significant.

To further analyze the association of the five *GP6* polymorphisms (rs1654410 T>C, rs1671153 T>G, rs1654419 G>A, rs12610286 A>G, and rs1654431 G>A) with RPL, we performed a combinatorial analysis (Table 3). Analysis of the five polymorphisms demonstrated that the frequency of *GP6* polymorphic genotype combinations was significantly higher in RPL patients compared to control participants. The genotype combination analysis of four *GP6* polymorphisms is also presented in Table 3 as *GP6* rs1654410/*GP6* rs1671153 TT/TT (AOR = 0.333 95% CI = 0.140–0.792; *p* = 0.013). Likewise, the two site combination genotypes *GP6* rs1654410/*GP6* rs1654419 TT/AA (AOR = 0.276 95% CI = 0.113–0.674; *p* = 0.005) and TT/GA (AOR = 0.202 95% CI = 0.050–0.813; *p* = 0.024) also demonstrated significant associations with RPL.

Since environmental factors are known to contribute to RPL, we also investigated the interaction of clinical characteristics and *GP6* genotypes. Logistic regression analysis was used for the association of each SNP with RPL risk adjusted by environmental factors. Table 4 shows that the *GP6* rs1654419 GG + GA genotype increased RPL risk when the following characteristics were present: platelet count of ≥242.11 × 10^3^ (AOR = 4.461; 95% CI = 2.234–8.905), follicle-stimulating hormone (FSH) level < 8.13 mIU/mL (AOR = 2.716; 95% CI = 1.458–5.057), and E2 level < 26.00 pg/mL (AOR = 2.322; 95% CI = 1.134–4.755). Thus, the *GP6* rs1654419 genotype may present an increased risk of RPL. However, these factors measured here undergo changes in the course of pregnancy.

Haplotype analyses were conducted to further assess the association of RPL occurrence with the five polymorphisms (Supplementary Table S2). Several haplotypes were found to be significantly associated with the incidence of RPL after FDR correction. Furthermore, the results from genotype combination analyses were significant for each allele, and these results were related to RPL incidence (Supplementary Table S3).

When the haplotype analysis was limited to four polymorphisms, we found that RPL was significantly associated with each allele (Supplementary Table S4). Limiting the haplotype analysis to three polymorphisms revealed that three variants (*GP6* rs1654410, *GP6* rs1671153, *GP6* rs1654419) were the leading genetic risk factors related to RPL (Supplementary Table S5).

Since clinical factors, including aPTT, PAI-1, PT, and homocysteine, have been associated with RPL, we investigated the association of these clinical variables with *GP6* gene variants in RPL patients who had suffered three or more pregnancy losses. We found that aPTT, PAI-1, PT, homocysteine, and PAI-1 levels were significantly associated with these RPL patients (Supplementary Tables S6–S8). Furthermore, Figure 1 depicts the analysis of variance for aPTT, PAI-1, PT, and homocysteine levels according to *GP6* polymorphisms (RPL ≥ 3). Our association data are statistically quite weak when comparing to the normal genome-wide association study (GWAS) significance threshold of 5 × 10^−8^.

## 4. Discussion

In this study, we selected five *GP6* polymorphisms (rs1654410 T>C, rs1671153 T>G, rs1654419 G>A, rs12610286 A>G, and rs1654431 G>A) that were candidate biomarkers for RPL risk.

Then, we investigated the association between these polymorphisms and RPL prevalence. We found that *GP6* polymorphisms were associated with an increased risk of RPL and that the *GP6* haplotypes rs1654410 T>C, rs1671153 T>G, rs1654419 G>A, rs12610286 A>G, and rs1654431 G>A occurred significantly more frequently in women with RPL. The haplotype analysis of the five genetic markers revealed that five haplotypes (*GP6* rs1654410 T>C, rs1671153 T>G, rs1654419 G>A, rs12610286 A>G, and rs1654431 G>A; [*p* < 0.001; FDR-corrected *p* < 0.001 OR, 19.13; 95% CI, 1.142–320.200; *p* = 0.0002; FDR-corrected *p* = 0.001] were significantly associated with increased RPL susceptibility.

Many *GP6* polymorphisms have been identified, and previous studies have demonstrated that genetic changes in *GP6* have an effect on platelet function. Platelets are small, acellular cells that aggregate to form blood clots on blood vessels and are essential for hemostasis. As a platelet membrane protein, *GP6* is generally accepted to be produced by platelet activation, adhesion, and aggregation. Interestingly, the importance of the genetic diversity of the *GP6* gene for platelet aggregation was highlighted by a genome-wide meta-analysis conducted by Johnson et al. [20]. Moreover, a previous study identified several SNPs (rs1671153, rs1654419, and rs1613662) in patients with sticky platelet syndrome (SPS) [21] and a history of miscarriage (rs1671152, rs1654433, rs1613662, rs1654416, and rs2304167) [22]. In addition to their normal role in hemostasis, platelets can cause arterial thrombosis by blocking the collateral arteries after a collagen-rich atherosclerotic plaque has ruptured, resulting in heart attack and stroke [23]. After an atherosclerotic plaque rupture, the relationship between *GP6* and thrombus *GP6*-specific signaling plays a major role in platelet adhesion to activated endothelium [24,25] and thrombus formation [26], whereas platelet *GP6* mediates adherence to the endothelial matrix [27].

*GP6* is a receptor that contains an immunoglobin-like domain, is structurally and functionally similar to an immunoreceptor, and is exclusively expressed in platelets and platelet precursors called megakaryocytes. Although it is unknown how thrombotic formation through GP6 can occur so rapidly, it is known that various agonists (collagen, thrombin, and ADP) activate GP6 on the platelet membrane to induce binding to fibrinogen and consequently promote the aggregation of platelets and granule release [28,29].

In addition, collagen activates platelets by promoting the phosphorylation of phospholipase C-γ2 (PLC-γ2) through *GP6* [30]. Platelets that circulate along the bloodstream respond to collagen that is exposed at the wound site on the vessel wall via *GP6* recognition and signaling. Collagen is a typical extracellular matrix that is used to form a thrombus, which is exposed to the bloodstream when a wound on the blood vessel wall develops, thus forming a platelet-attachable surface in the artery and acting as the main stimulant for platelet activation. Therefore, achieving a better understanding of this process may lead to the development of treatment modalities that inhibit or prevent thrombus formation. We predict that these mechanisms will be important in RPL patients based on the association with *GP6*.

*GP6* dimers recognize collagen, and a portion of *GP6* is present as a dimer in resting platelets. Upon platelet activation, *GP6* dimer levels increase [31,32]. *GP6* forms a noncovalent bond with platelet glycoprotein Ibα (GPIbα) on activated platelets and signals through the associated proto-oncogene tyrosine-protein kinase Src (Src)/spleen tyrosine kinase (Syk) kinase pathway. Crucially important in the *GP6*-mediated signal is the presence of an immunoreceptor tyrosine-based activation motif (ITAM) [33,34,35] in the cytoplasmic domain Fc receptor common γ signaling chain (FcRγ). Interestingly, *GP6* signaling not only regulates normal hemostasis but also contributes to cardiovascular disease, inflammation, coagulation control, and tumor metastasis [36,37,38,39,40,41].

Currently, there are some reported associations between *GP6* and platelet syndrome in RPL [16,34,42,43].

In the present study, polymorphic *GP6* genotype frequencies rose significantly as the frequency of pregnancy loss increased. Moreover, *GP6* genotypes and haplotypes were associated with known contributors to increased blood coagulation, including elevated plasma PAI-1 and BMI, lower PT and aPTT, and plasma concentrations of vascular risk factors (homocysteine, FSH, and total cholesterol) in RPL. We anticipate that these *GP6* genotype combinations contribute to the incidence of RPL in Korean women. Alterations in the fibrinolysis cascade that cause hypo-fibrinization or hyperfibrinolysis may interfere with the placenta and result in poor pregnancy outcomes. Defects in this process can negatively affect trophoblast transplantation as well as the placenta and may result in RPL. Moreover, higher PLT indices have been reported to increase the risk of thrombosis. Previous reports also suggest that hormones, including FSH, LH, and E2 are involved in RPL, since FSH, LH, and E2 levels were elevated in the RPL group compared with the control group. The control group consisted of women who had abortions with known causes, such as uterine septum and parental chromosomal abnormalities. However, in hemostasis, factor V Leiden mutation, prothrombin G20210A variant, the decreased activity of antithrombin, protein C, protein S, the increased coagulation factor VIII activity, dysfibrinogenaemia, fibrinolysis abnormalities, antiphospholipid syndrome, and the detection of plasma levels of substances released from platelets factors related to thrombophilia should also be investigated.

Reports on the relationship between GP6 and pregnancy loss are very limited. In addition, the biological function of GP6 pregnancy is not well known. Genetic mutations in the blood coagulation factor cause a prethrombotic condition through deficiencies of blood coagulation inhibitors, the overproduction of pre-coagulation proteins, abnormalities in fibrinolysis, and damage to the vascular endothelium. As the diagnosis of neonatal thromboembolism increases, the role of genetic risk factors becomes more important.

GP6 is a platelet transmembrane glycoprotein that plays a significant role in collagen-initiated signal transduction and platelet pro-coagulant activity; therefore, the observed variation in the GP6 gene region may influence risk for thromboembolic disorders [44].

A previous report shows that a significantly higher occurrence of mutant genotypes of GP6 SNPs, namely rs1671153, rs1654410, rs1654419, and rs1613662, in recurrent miscarriages (RM) cases, suggesting a risk association for pregnancy loss [45]. Another study has shown risk associations of mutant genotypes at rs1671153, rs1654419, and rs1613662 SNPs with thrombotic disorders [46].

Patients of pregnancy loss demonstrate reduced platelet function [47]. The GP6 plays a critical role in platelet adhesion and activation, and GP6 has a major role in collagen-induced platelet signaling [45]. It is thought that changes in platelet adhesion and activation by GP6 polymorphism (rs1654410T>C, rs1654419 G>A, and rs1654431 G>A) would have influenced platelet reactivity toward collagen and therefore influence platelet function and the maintenance of pregnancy.

Based on the present study, we suggest that the genotype combinations of GP6 polymorphisms rs1654410/GP6 rs1671153 (CC/TT) may contribute to the diagnosis of RPL in Korean women. Therefore, additional studies are needed to clarify the association between *GP6* polymorphisms and RPL.

MicroRNAs (miRNAs) are associated with platelet reactivity; however, there is a lack of information regarding the role of miRNAs in megakaryocyte signaling cascades, and miRNAs are not known to regulate collagen-induced *GP6* signaling. One previous report bioinformatically predicted that miR-15a-5p targets were expressed in megakaryocytes and subsequently enriched target genes that are also known to be downstream targets of platelet *GP6* signaling [48]. These findings indicate that miR-15a-5p regulates the potential master-miRNA *GP6*-mediated megakaryocyte signaling and platelet activation. Therefore, the present study provides a basis for future research associated with miRNA in RPL patients.

It is not sufficient to make recommendations for RPL patient management based on our research results alone. Our results indicate that several polymorphisms are associated with clinical variables in RPL patients: GP6 rs1671153 T>G, rs1654419 G>A, and rs12610286 A>G were associated with higher homocysteine levels, elevated creatinine levels, and PAI-1, respectively. Therefore, GP6 polymorphisms may contribute to RPL and are potential biomarkers for assessing RPL risk. However, our study does have various limitations, which are outlined in the Discussion, and efforts to overcome these problems are necessary. Our chosen method of liquid biopsy has been recently investigated in depth, and we therefore plan to continue research to prevent platelet-related diseases by analyzing GP6-related microRNA, non-coding RNAs, and exosomes; in this regard, the present study provides a basis for future research associated with miRNA in RPL patients.

Although we found an association between GP6 polymorphisms and RPL, there are some limitations to our study: lack of a survey for vascular risk factors, clinical insufficiency from control participants, and the relatively small sample size of the control and RPL group, which highlights the need for additional studies in larger patient populations. Mechanism by which *GP6* polymorphisms affect the onset of RPL remains unclear. Additional information regarding risk factors for RPL patients is lacking, and additional research is needed. The population of this study was restricted to Korean patients.

## 5. Conclusions

In conclusion, we identified associations between *GP6* gene polymorphisms (rs1654410 T>C, rs1671153 T>G, rs1654419 G>A, rs12610286 A>G, and rs1654431 G>A) and RPL prevalence in Korean women. Our findings suggest that *GP6* polymorphisms may contribute to RPL and are potential biomarkers for assessing RPL risk. For example, several polymorphisms were associated with clinical variables in RPL patients: *GP6* rs1671153 T>G, rs1654419 G>A, and rs12610286 A>G were associated with higher homocysteine levels, elevated creatinine levels, and PAI-1, respectively.

Moreover, the haplotype frequencies of *GP6* rs1654410 T>C, rs1671153 T>G, rs1654419 G>A, rs12610286 A>G, and rs1654431 G>A were significantly different between RPL patients and control participants. Together, these results highlight the need for large and heterogeneous genetic studies to confirm the present findings and to validate potential biomarkers of RPL for prevention and prognosis.

## Figures and Tables

**Table 1:** Clinical variables in control participants and RPL patients.

Characteristics	Controls (*n* = 219)	RPL (*n* = 388)	*p*
Age (years)	32.75 ± 3.84	33.20 ± 4.54	0.211
BMI (kg/m^2^)	21.63 ± 3.44	19.77 ± 6.83	0.291
Previous pregnancy losses (*n*)	N/A	3.28 ± 1.83	
Live births (*n*)	1.71 ± 0.58	N/A	
Mean gestational age (weeks)	39.14 ± 1.56	7.32 ± 2.05	<0.0001
Homocysteine (µmol/L)	35.98 ± 4.13	37.30 ± 3.36	
Total cholesterol (mg/dL)	N/A	187.73 ± 49.41	
Uric acid (mg/dL)	N/A	3.80 ± 0.83	0.357
PLT (10^3^/µL)	242.11 ± 61.86	255.43 ± 59.22	0.034
PT (sec)	0.84 ± 0.09	0.98 ± 0.09	<0.0001
PAI-1 (ng/mL)	NA	10.37 ± 5.70	
aPTT (sec)	33.05 ± 3.08	32.23 ± 4.32	0.203
BUN (mg/dL, mean ±SD)	N/A	9.98 ± 2.76	
Creatinine (mg/dL, mean ±SD)	N/A	0.72 ± 0.12	
FSH (mIU/mL, mean ±SD)	8.13 ± 2.80	7.51 ± 10.52	0.546
LH (mIU/mL, mean ±SD)	3.38 ± 1.87	6.29 ± 12.08	0.012
E2 (pg/mL, mean ±SD)	26.00 ± 14.94	35.70 ± 29.45	0.001

Note: RPL, recurrent pregnancy loss; BMI, body mass index; PLT, platelet count; PT, prothrombin time; PAI-1, plasminogen activator inhibitor-1; aPTT, activated par tial thromboplastin time; BUN, blood urea nitrogen; FSH, follicle-stimulating hormone; LH, luteinizing hormone; E2, estradiol.

**Table 2:** Genotype frequencies of glycoprotein 6 (*GP6*) polymorphisms in the control and RPL patients.

Genotypes	Controls (*n* = 219)	PL ≥ 2 (*n* = 388)	AOR (95% CI)	*p*	*FDR-P*	Controls (*n* = 219)	PL ≥ 3 (*n* = 138)	AOR (95% CI)	*p*	*FDR-P*	PL ≥ 4 (*n* = 74)	AOR (95% CI)	*p*
*GP6* rs1654410T>C													
TT	88 (40.2)	189 (48.7)	1.000 (reference)			88 (40.2)	69 (50.0)	1.000 (reference)			37 (50.0)	1.000 (reference)	
TC	108 (49.3)	176 (45.4)	1.218 (0.737–2.012)	0.432	0.781	108 (49.3)	64 (46.4)	0.760 (0.488–1.183)	0.223	0.309	34 (45.9)	0.760 (0.440–1.312)	0.325
CC	23 (10.5)	23 (5.9)	1.285 (0.693–2.385)	0.446	0.511	23 (10.5)	5 (3.6)	0.292 (0.105–0.815)	0.019	0.095	3 (4.1)	0.338 (0.094–1.209)	0.095
Dominant (TT vs. TC + CC)			1.236 (0.766–1.995)	0.355	0.627			0.684 (0.444–1.054)	0.085	0.142		0.695 (0.408–1.185)	0.182
Recessive (TT + TC vs. CC)			1.126 (0.672–1.888)	0.641	0.641			0.348 (0.128–0.944)	0.038	0.190		0.395 (0.114–1.368)	0.143
HWE *P*	0.423	0.302											
*GP6* rs1671153 T>G													
TT	107 (48.9)	216 (55.7)	1.000 (reference)			107 (48.9)	74 (53.6)	1.000 (reference)			37 (50.0)	1.000 (reference)	
TG	98 (44.7)	155 (39.9)	0.788 (0.558–1.113)	0.176	0.781	98 (44.7)	55 (39.9)	0.826 (0.528–1.291)	0.401	0.401	29 (39.2)	0.879 (0.501–1.543)	0.653
GG	14 (6.4)	17 (4.4)	0.625 (0.295–1.324)	0.220	0.511	14 (6.4)	9 (6.5)	1.039 (0.421–2.567)	0.934	0.934	8 (10.8)	1.905 (0.720–5.042)	0.194
Dominant (TT vs. TG + GG)			0.770 (0.551–1.076)	0.125	0.625			0.852 (0.554–1.310)	0.465	0.465		0.994 (0.584–1.693)	0.983
Recessive (TT + TG vs. GG)			0.693 (0.334–1.439)	0.325	0.542			1.153 (0.480–2.768)	0.751	0.751		2.051 (0.810–5.195)	0.130
HWE *P*	0.173	0.097											
*GP6* rs1654419 G>A													
GG	122 (55.7)	262 (67.5)	1.000 (reference)			122 (55.7)	96 (69.6)	1.000 (reference)			54 (73.0)	1.000 (reference)	
GA	77 (35.2)	106 (27.3)	0.920 (0.580–1.461)	0.625	0.781	77 (35.2)	36 (26.1)	0.607 (0.375–0.982)	0.042	0.118	17 (23.0)	0.506 (0.273–0.940)	0.031
AA	20 (9.1)	20 (5.2)	1.294 (0.700–2.394)	0.511	0.511	20 (9.1)	6 (4.3)	0.392 (0.151–1.018)	0.055	0.114	3 (4.1)	0.345 (0.098–1.213)	0.097
Dominant (GG vs. GA + AA)			1.006 (0.653–1.551)	0.878	0.878			0.563 (0.358–0.885)	0.013	0.055		0.473 (0.265–0.846)	0.012
Recessive (GG + GA vs. AA)			1.355 (0.774–2.372)	0.288	0.542			0.464 (0.181–1.189)	0.110	0.270		0.427 (0.123–1.486)	0.181
HWE *P*	0.085	0.642											
*GP6* rs12610286 A>G													
AA	118 (53.9)	202 (52.1)	1.000 (reference)			118 (53.9)	64 (46.4)	1.000 (reference)			31 (41.9)	1.000 (reference)	
AG	90 (41.1)	148 (38.1)	0.979 (0.691–1.387)	0.905	0.905	90 (41.1)	61 (44.2)	1.305 (0.832–2.049)	0.247	0.309	38 (51.4)	1.725 (0.987–3.013)	0.056
GG	11 (5.0)	38 (9.8)	2.025 (0.995–4.120)	0.052	0.260	11 (5.0)	13 (9.4)	2.106 (0.888–4.994)	0.091	0.114	5 (6.8)	1.670 (0.538–5.187)	0.375
Dominant (AA vs. AG + GG)			1.088 (0.780–1.517)	0.621	0.776			1.384 (0.899–2.129)	0.140	0.175		1.689 (0.986–2.895)	0.056
Recessive (AA + AG vs. GG)			2.006 (1.001–4.018)	0.050	0.250			1.822 (0.787–4.219)	0.162	0.270		1.267 (0.423–3.803)	0.673
HWE *P*	0.238	0.160											
*GP6* rs1654431 G>A													
GG	79 (36.1)	154 (39.7)	1.000 (reference)			79 (36.1)	34 (24.6)	1.000 (reference)			18 (24.3)	1.000 (reference)	
GA	104 (47.5)	178 (45.9)	0.875 (0.608–1.259)	0.472	0.781	104 (47.5)	74 (53.6)	1.663 (1.007–2.746)	0.047	0.118	45 (60.8)	1.967 (1.052–3.677)	0.034
AA	36 (16.4)	56 (14.4)	0.774 (0.468–1.279)	0.318	0.511	36 (16.4)	30 (21.7)	1.772 (0.932–3.369)	0.081	0.114	11 (14.9)	1.391 (0.582–3.325)	0.458
Dominant (GG vs. GA + AA)			0.856 (0.608–1.207)	0.376	0.627			1.746 (1.082–2.818)	0.022	0.055		1.856 (1.013–3.400)	0.045
Recessive (GG + GA vs. AA)			0.833 (0.526–1.319)	0.436	0.545			1.309 (0.757–2.263)	0.336	0.420		0.860 (0.412–1.799)	0.689
HWE *P*	0.857	0.694											

Note: AORs were adjusted for age of participants. RPL, recurrent pregnancy loss; AOR, adjusted odds ratio; CI, confidence interval; *FDR-P*, false discovery rate-adjusted *p*-value.

**Table 3:** Genotype combinations of GP6 polymorphisms in control participants and (RPL) patients.

Genotypes	Controls (*n* = 219)	RPL Patients (*n* = 388)	AOR (95% CI)	*p ^a^*	*FDR-P ^b^*
GP6 rs1654410/GP6 rs1671153					
TT/TT	53 (24.2)	107 (27.6)	1.000 (reference)		
TT/TG	43 (19.6)	61 (15.7)	0.687 (0.411–1.146)	0.150	0.750
CC/TT	15 (6.8)	10 (2.6)	0.333 (0.140–0.792)	0.013	0.039
CC/GG	2 (0.9)	1 (0.3)	0.254 (0.022–2.873)	0.268	0.402
GP6 rs1654410/GP6 rs1654419					
TT/GG	51 (23.3)	109 (28.1)	1.000 (reference)		
TT/AA	15 (6.8)	10 (2.6)	0.276 (0.113–0.674)	0.005	0.015
CC/GG	15 (6.8)	19 (4.9)	0.662 (0.307–1.430)	0.294	0.725
CC/GA	7 (3.2)	3 (0.8)	0.202 (0.050–0.813)	0.024	0.048
CC/AA	1 (0.5)	1 (0.3)	0.443 (0.027–7.270)	0.569	0.569
GP6 rs1654410/GP6 rs12610286					
TT/AA	48 (21.9)	100 (25.8)	1.000 (reference)		
TT/AG	48 (21.9)	57 (14.7)	0.570 (0.339–0.957)	0.033	0.132
CC/AG	11 (5.0)	10 (2.6)	0.451 (0.176–1.153)	0.096	0.243
CC/GG	12 (5.5)	13 (3.4)	0.540 (0.228–1.280)	0.162	0.243
GP6 rs1654410/GP6 rs1654431					
TT/GG	35 (16.0)	74 (19.1)	1.000 (reference)		
TT/GA	57 (26.0)	78 (20.1)	0.640 (0.377–1.087)	0.099	0.396
CC/GG	10 (4.6)	6 (1.5)	0.282 (0.094–0.852)	0.025	0.100
GP6 rs1671153/GP6 rs1654419					
TT/GG	73 (33.3)	142 (36.6)	1.000 (reference)		
TG/AA	12 (5.5)	10 (2.6)	0.391 (0.157–0.974)	0.044	0.132
GG/GA	6 (2.7)	5 (1.3)	0.444 (0.131–1.511)	0.194	0.291
GP6 rs1671153/GP6 rs12610286					
TT/AA	53 (24.2)	122 (31.4)	1.000 (reference)		
TT/AG	52 (23.7)	66 (17.0)	0.556 (0.342–0.905)	0.018	0.072
TG/AG	44 (20.1)	59 (15.2)	0.604 (0.362–1.007)	0.053	0.106
GP6 rs1671153/GP6 rs1654431					
TT/GG	56 (25.6)	85 (21.9)	1.000 (reference)		
TT/AA	6 (2.7)	33 (8.5)	3.476 (1.360–8.885)	0.009	0.036
TG/GG	29 (13.2)	54 (13.9)	1.224 (0.697–2.151)	0.482	0.699
GP6 rs1654419/GP6 rs12610286					
GG/AA	69 (31.5)	133 (34.3)	1.000 (reference)		
AA/GG	7 (3.2)	29 (7.5)	2.065 (0.857–4.977)	0.106	0.265
GA/AG	36 (16.4)	41 (10.6)	0.594 (0.348–1.014)	0.056	0.265
GA/AG	11 (5.0)	4 (1.0)	0.181 (0.055–0.595)	0.005	0.015
GP6 rs1654419/GP6 rs1654431					
GG/GG	58 (26.5)	92 (23.7)	1.000 (reference)		
AA/AA	13 (5.9)	49 (12.6)	2.203 (1.091–4.447)	0.028	0.112
AA/AA	7 (3.2)	17 (4.4)	1.535 (0.600–3.931)	0.371	0.543
GP6 rs12610286/GP6 rs1654431					
AA/GG	44 (20.1)	78 (20.2)	1.000 (reference)		
GG/AA	8 (3.7)	34 (8.8)	2.373 (1.004–5.608)	0.049	0.196
AG/GA	51 (23.3)	62 (16.0)	0.685 (0.406–1.156)	0.157	0.314

Note: RPL, recurrent pregnancy loss; AOR, adjusted odds ratio; CI, confidence interval; a, Fisher’s exact test; b, FDR-adjusted *p* value.

**Table 4:** Interaction of RPL incidence and environmental factors.

Characteristics	GP6 rs1654410 (TT vs. TC + CC)		GP6 rs1671153 (TT vs. TG + GG)		GP6 1654419 (GG + GA vs. AA)		GP6 rs12610286 (AA + AG vs. GG)		GP6 rs1654431 (GG + GA vs. AA)	
	AOR (95% CI) *	*p*	AOR (95% CI) *	*p*	AOR (95% CI) *	*p*	AOR (95% CI) *	*p*	AOR (95% CI) *	*p*
**Age**										
<32	1.389 (0.810–2.383)	0.233	0.944 (0.551–1.617)	0.833	1.672 (0.950–2.944)	0.075	0.888 (0.518–1.520)	0.664	1.291 (0.747–2.229)	0.360
≥32	0.789 (0.516–1.207)	0.274	1.280 (0.835–1.961)	0.258	1.151 (0.738–1.795)	0.536	0.908 (0.595–1.384)	0.653	1.156 (0.749–1.783)	0.513
BMI										
<25 kg/m^2^	1.325 (0.798–2.200)	0.277	0.821 (0.495–1.362)	0.445	0.860 (0.520–1.423)	0.558	1.320 (0.795–2.193)	0.284	0.784 (0.463–1.328)	0.365
≥25 kg/m^2^	0.434 (0.099–1.893)	0.267	1.433 (0.365–5.623)	0.606	2.398 (0.544–10.571)	0.248	0.487 (0.118–2.006)	0.319	1.245 (0.307–5.041)	0.759
Platelet										
<242.11 × 10^3^ cell	0.908 (0.498–1.655)	0.752	1.078 (0.586–1.981)	0.810	1.056 (0.578–1.931)	0.859	0.841 (0.462–1.532)	0.572	1.294 (0.710–2.356)	0.400
≥242.11 × 10^3^ cell	1.108 (0.622–1.973)	0.728	1.267 (0.711–2.258)	0.422	4.461 (2.234–8.905)	0.0001	0.691 (0.389–1.225)	0.205	1.184 (0.657–2.133)	0.575
PT										
≥0.84 s	1.447 (0.314–6.666)	0.635	0.596 (0.114–3.127)	0.541	1.897 (0.389–9.251)	0.429	0.335 (0.056–2.006)	0.231	0.803 (0.170–3.782)	0.781
<0.84 s	1.208 (0.533–2.740)	0.651	0.630 (0.274–1.448)	0.277	0.696 (0.306–1.582)	0.387	0.844 (0.372–1.913)	0.684	0.761 (0.314–1.843)	0.545
aPTT										
<33.05 s	1.074 (0.486–2.370)	0.861	0.598 (0.265–1.350)	0.216	1.272 (0.567–2.852)	0.559	0.963 (0.435–2.132)	0.926	1.061 (0.463–2.432)	0.890
≥33.05 s	1.754 (0.664–4.630)	0.257	0.723 (0.272–1.924)	0.516	0.915 (0.349–2.396)	0.856	0.781 (0.299–2.043)	0.615	0.711 (0.259–1.950)	0.507
FSH										
<8.13 mIU/mL	1.013 (0.579–1.772)	0.965	0.833 (0.479–1.447)	0.516	2.716 (1.458–5.057)	0.002	0.685 (0.393–1.192)	0.181	0.950 (0.531–1.700)	0.862
≥8.13 mIU/mL	0.906 (0.382–2.150)	0.822	0.672 (0.283–1.595)	0.367	0.921 (0.381–2.226)	0.855	0.880 (0.365–2.121)	0.776	2.842 (0.857–9.424)	0.088
LH										
<3.38 mIU/mL	1.427 (0.702–2.901)	0.326	1.195 (0.590–2.420)	0.622	2.047 (0.963–4.351)	0.063	0.731 (0.361–1.482)	0.385	0.764 (0.359–1.628)	0.485
≥3.38 mIU/mL	0.713 (0.370–1.374)	0.312	0.540 (0.284–1.025)	0.060	1.707 (0.883–3.298)	0.112	0.802 (0.421–1.527)	0.502	1.311 (0.642–2.677)	0.457
E2										
<26.00 pg/mL	1.439 (0.720–2.880)	0.303	0.685 (0.342–1.372)	0.285	2.322 (1.134–4.755)	0.021	0.510 (0.247–1.050)	0.068	0.874 (0.400–1.911)	0.736
≥26.00 pg/mL	0.795 (0.404–1.563)	0.506	0.840 (0.429–1.647)	0.612	1.821 (0.905–3.664)	0.093	0.506 (0.256–1.002)	0.051	1.604 (0.785–3.280)	0.195

Note: AOR, adjusted odds ratio; 95% CI, 95% confidence interval; BMI, body mass index; PLT, platelet count; PT, prothrombin time; aPTT, activated par tial thromboplastin time; FSH, follicle stimulating hormone; LH, luteinizing hormone; E2, estradiol. The adjusted odds ratio on the basis of risk factors, such as age, BMI, PLT, PT, aPTT, FSH, LH, and E2.

## Supplementary Materials

The following are available online at https://www.mdpi.com/2073-4425/11/8/862/s1. Figure S1. Linkage disequilibrium (LD) patterns of GP6 SNPs. Values in squares are LD between single markers. There were no strong LDs between loci rs1654410C>T, rs1671153 T>G, rs1654419 G>A, rs12610286 A>G and rs1654431 G> in RPL subjects. Table S1: Information of GP6 gene polymorphisms for PCR-RFLP and real-time PCR analysis. Table S2: Haplotype analysis of GP6 gene polymorphisms in RPL and controls. Table S3: Genotype combination for the GP6 polymorphisms in recurrent pregnancy loss (RPL) patients and control subjects. Tables S4–S5: Haplotype analysis of GP6 gene polymorphisms in RPL and controls. Tables S6–S8: Differences of various clinical parameters according to GP6 gene polymorphisms in RPL. Table S9: Frequencies of GP6 polymorphisms in world-wide populations.
