# Supplementary material for: Association between Platelet-Specific Collagen Receptor Glycoprotein 6 Gene Variants, Selected Biomarkers, and Recurrent Pregnancy Loss in Korean Women
Source: Genes (Basel). 2020 Jul 29;11(8):862. doi: 10.3390/genes11080862 (PMC7464359; doi:10.3390/genes11080862)
Supplement: Supplementary file 1 [file genes-11-00862-s001.pdf]

Article

Association between Platelet-Specific Collagen Receptor Glycoprotein 6 Gene Variants, Selected Biomarkers, and Recurrent Pregnancy Loss in Korean Women

Hui Jeong An[1,†], Eun Hee Ahn[2,†], Jung Oh Kim[1], Chang Soo Ryu[1], Han Sung Park[1], Sung Hwan Cho[1], Ji Hyang Kim[2], Woo Sik Lee[3], Jung Ryeol Lee[4], Young Ran Kim[2,*] and Nam Keun Kim[1,*]

Supplementary Files

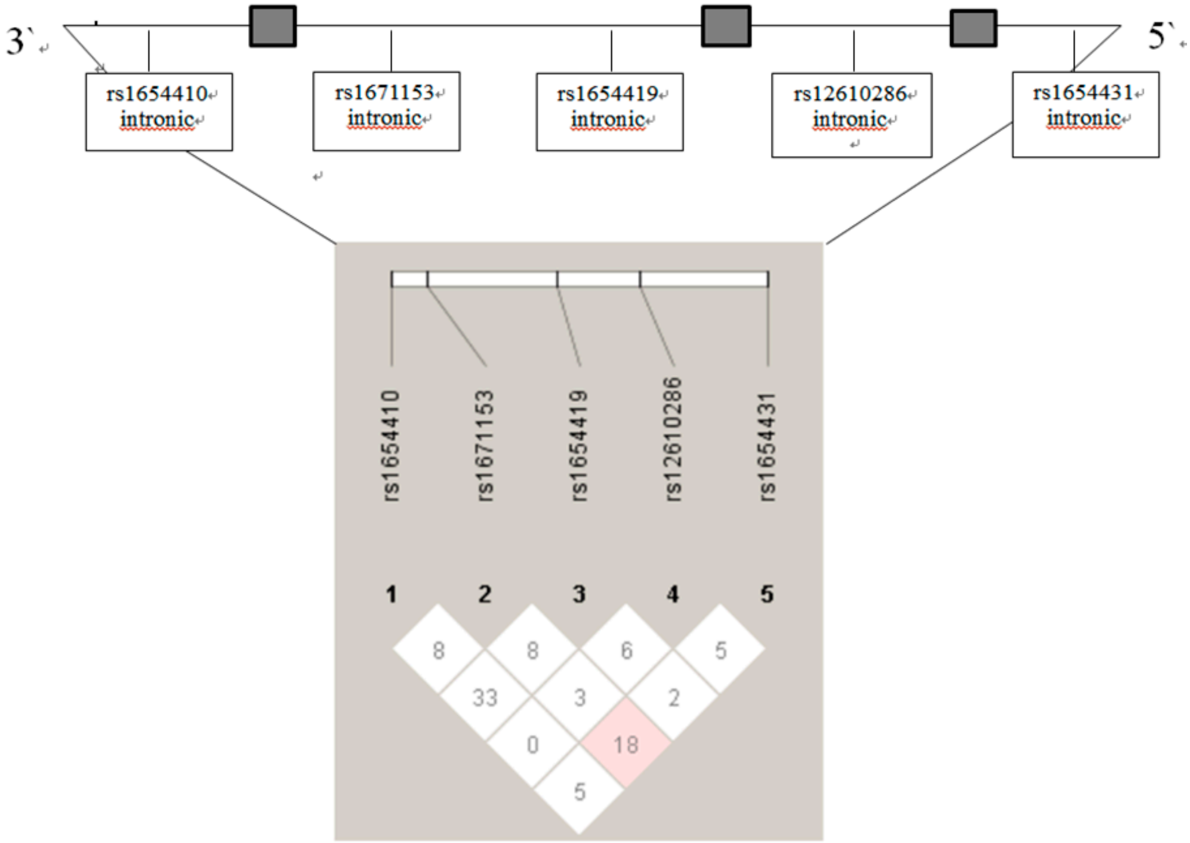

Figure S1. Linkage disequilibrium (LD) patterns of GP6 SNPs. Values in squares are LD between single markers. There were no strong LDs between loci rs1654410C>T, rs1671153 T>G, rs1654419 G>A, rs12610286 A>G and rs1654431 G> in RPL subjects.

Table S1. Information of *GP6* gene polymorphisms for PCR-RFLP and real-time PCR analysis.

Polymorphism	Rs number	Primer sequence	Probe sequence	Annealing temperature	Restriction enzyme
GP6T>C	rs1654410	Forward 5`-AGTACCTGAGAGTAGGCATGTGG-3`<br>Reverse 5`-CTCCAAATGTAAGAATGGGTCAA-3`	-	60℃	*Mbo* II
GP6T>G	rs1671153	Forward 5`-CTTTGGACTGGCGGTGAT-3`<br>Reverse 5`-CGGCCCATGCTTTTCTTA-3`	-	58℃	*Hph* I
GP6G>A	rs1654419	Forward 5`-GGCTCTTTCCAGCATGTTTC-3`<br>Reverse 5`-TTGTGTGAGGCGTGTTATCC-3`	-	59℃	*Cse* I
GP6A>G	rs12610286	Forward 5`- GTGAGGCACTCTCCTGG -3`<br>Reverse 5`- GGCTCAAGTGGTCCTC -3`	A: 5`-FAM- CCAAACAATTAACaTATTTGAAAA -3`<br>C: 5`-JOE- CCAAACAATTAACgTATTTGAAAA -3`-	57℃	
GP6G>A	rs1654431	Forward 5`-GGCTCAGGATGTGATGAGG -3`<br>Reverse 5`-GTGAAGTCATATCTCACTG -3`	A: 5`-FAM- AGCCGTAGCCaGCTCCTCA -3`<br>C: 5`-JOE- AGCCGTAGCCgGCTCCTCA -3`	63℃	-

Note: PCR-RFLP, polymorphism chain reaction-restriction fragment length polymorphism

Table S2. Haplotype analysis of *GP6* gene polymorphisms in RPL and controls.

Haplotypes	Control (n= 438)	Case (n=776)	OR(95% CI)	P^a	$FDR\text{-}P^b$
GP6 rs1654410 /GP6 rs1671153 /GP6 rs1654419 /GP6 rs12610286 /GP6 rs1654431					
T-T-G-A-G	74 (16.8)	167 (21.5)	1.000 (reference)		
T-T-G-A-A	35 (7.9)	81 (10.4)	1.025 (0.633 - 1.660)	1.000	1.000
T-T-G-G-G	32 (7.4)	55 (7.1)	0.762 (0.455 - 1.274)	0.349	0.524
T-T-G-G-A	17 (3.9)	36 (4.7)	0.938 (0.496 - 1.777)	0.870	0.870
T-T-A-A-G	32 (7.3)	47 (6.0)	0.651 (0.385 - 1.101)	0.130	0.390
T-T-A-A-A	5 (1.2)	13 (1.6)	1.152 (0.396 - 3.350)	1.000	1.000
T-T-A-G-G	12 (2.9)	10 (1.3)	0.369 (0.153 - 0.893)	0.031	0.052
T-T-A-G-A	8 (1.9)	12 (1.6)	0.665 (0.261 - 1.694)	0.453	0.566
T-G-G-A-G	30 (6.9)	20 (2.6)	0.295 (0.158 - 0.554)	0.0002	0.001
T-G-G-A-A	17 (3.9)	31 (4.0)	0.808 (0.421 - 1.551)	0.610	0.610
T-G-G-G-G	0 (0.0)	21 (2.8)	19.13 (1.142 - 320.200)	0.002	0.005
T-G-G-G-A	5 (1.3)	10 (1.3)	0.886 (0.293 - 2.684)	0.781	0.781
T-G-A-A-G	8 (1.9)	23 (3.0)	1.274 (0.545 - 2.981)	0.680	0.781
T-G-A-A-A	9 (2.1)	13 (1.7)	0.640 (0.262 - 1.563)	0.343	0.781
T-G-A-G-G	0 (0.0)	1 (0.2)	1.334 (0.054 - 33.160)	1.000	1.000
T-G-A-G-A	12 (2.6)	0 (0.0)	0.018 (0.001 - 0.305)	<0.0001	0.0002
C-T-G-A-G	56 (12.9)	52 (6.7)	0.412 (0.258 - 0.656)	0.0002	0.0002
C-T-G-A-A	22 (5.0)	48 (6.2)	0.967 (0.544 - 1.717)	1.000	1.000
C-T-G-G-G	13 (3.1)	22 (2.8)	0.750 (0.358 - 1.569)	0.442	0.524
C-T-G-G-A	0 (0.0)	12 (1.6)	11.12 (0.649 - 190.400)	0.021	0.052
C-T-A-A-G	5 (1.2)	7 (0.9)	0.620 (0.191 - 2.019)	0.524	0.524
C-T-A-A-A	0 (0.0)	19 (2.4)	17.35 (1.033 - 291.300)	0.002	0.010
C-T-A-G-G	3 (0.8)	0 (0.0)	0.064 (0.003 - 1.247)	0.031	0.052
C-T-A-G-A	4 (1.0)	0 (0.0)	0.049 (0.003 - 0.930)	0.010	0.040
C-G-G-A-G	10 (2.2)	15 (2.0)	0.665 (0.285 - 1.549)	0.369	0.633
C-G-G-A-A	6 (1.4)	20 (2.5)	1.477 (0.570 - 3.830)	0.504	0.633
C-G-G-G-G	8 (1.7)	14 (1.8)	0.775 (0.312 - 1.928)	0.633	0.633
C-G-G-G-A	7 (1.6)	15 (1.9)	0.950 (0.372 - 2.426)	1.000	1.000
C-G-A-A-G	0 (0.0)	10 (1.3)	9.34 (0.540 - 161.600)	0.036	0.036
C-G-A-A-A	4 (0.9)	0 (0.0)	0.049 (0.003 - 0.930)	0.010	0.020
C-G-A-G-G	0 (0.0)	0 (0.0)	-	-	-
C-G-A-G-A	2 (0.6)	2 (0.3)	0.443 (0.061 - 3.208)	0.590	0.590

Note: ORs and 95% CIs of each allele combinations were calculated with reference to frequencies of all others using Fisher's exact test. *P* value by Fisher's exact test.; OR = odds ratio; CI = confidence interval. [a] Fisher's exact test; [b] *FDR*-adjusted *P* value.

Table S3. Genotype combination for the *GP6* polymorphisms in recurrent pregnancy loss (RPL) patients and control subjects.

Genotypes	Controls (n=219)	RPL patients (n=388)	AOR (95% CI)	*P*[a]	*FDR-P*[b]
***GP6* rs1654410 /rs1671153**					
TT/TT	53 (24.2)	107 (27.6)	1.000 (reference)		
TT/TG	43 (19.6)	61 (15.7)	0.687 (0.411 - 1.146)	0.150	0.750
TT/GG	5 (2.3)	8 (2.1)	0.803 (0.250 - 2.579)	0.712	0.995
TC/TT	45 (20.5)	93 (24.0)	1.004 (0.617 - 1.634)	0.988	0.995
TC/TG	45 (20.5)	86 (22.2)	0.944 (0.579 - 1.538)	0.816	0.995
TC/GG	5 (2.3)	10 (2.6)	0.996 (0.321 - 3.091)	0.995	0.995
CC/TT	15 (6.8)	10 (2.6)	0.333 (0.140 - 0.792)	0.013	0.039
CC/TG	6 (2.7)	12 (3.1)	1.038 (0.362 - 2.973)	0.945	0.945
CC/GG	2 (0.9)	1 (0.3)	0.254 (0.022 - 2.873)	0.268	0.402
GP6 rs1654410 /rs1654419					
TT/GG	51 (23.3)	109 (28.1)	1.000 (reference)		
TT/GA	35 (16.0)	57 (14.7)	0.762 (0.446 - 1.303)	0.32	0.480
TT/AA	15 (6.8)	10 (2.6)	0.276 (0.113 - 0.674)	0.005	0.015
TC/GG	66 (30.1)	124 (32.0)	0.874 (0.559 - 1.368)	0.557	0.557
TC/GA	26 (11.9)	55 (14.2)	0.969 (0.545 - 1.725)	0.915	0.915
TC/AA	3 (1.4)	10 (2.6)	1.615 (0.424 - 6.151)	0.483	0.725
CC/GG	15 (6.8)	19 (4.9)	0.662 (0.307 - 1.430)	0.294	0.725
CC/GA	7 (3.2)	3 (0.8)	0.202 (0.050 - 0.813)	0.024	0.048
CC/AA	1 (0.5)	1 (0.3)	0.443 (0.027 - 7.270)	0.569	0.569
GP6 rs1654410 /rs12610286					
TT/AA	48 (21.9)	100 (25.8)	1.000 (reference)		
TT/AG	48 (21.9)	57 (14.7)	0.570 (0.339 - 0.957)	0.033	0.132
TT/GG	5 (2.3)	19 (4.9)	1.818 (0.639 - 5.175)	0.263	0.351
TC/AA	46 (21.0)	105 (27.1)	1.105 (0.678 - 1.803)	0.689	0.689
TC/AG	43 (19.6)	65 (16.8)	0.736 (0.439 - 1.235)	0.246	0.351
TC/GG	6 (2.7)	19 (4.9)	1.533 (0.575 - 4.087)	0.393	0.393
CC/AA	11 (5.0)	10 (2.6)	0.451 (0.176 - 1.153)	0.096	0.243
CC/AG	12 (5.5)	13 (3.4)	0.540 (0.228 - 1.280)	0.162	0.243
CC/GG	0 (0.0)	0 (0.0)	-	-	-
GP6 rs1654410 /rs1654431					
TT/GG	35 (16.0)	74 (19.1)	1.000 (reference)		
TT/GA	57 (26.0)	78 (20.1)	0.640 (0.377 - 1.087)	0.099	0.396
TT/AA	9 (4.1)	24 (6.2)	1.238 (0.516 - 2.970)	0.633	0.844
TC/GG	43 (19.6)	65 (16.8)	0.714 (0.409 - 1.248)	0.238	0.476
TC/GA	41 (18.7)	83 (21.4)	0.951 (0.548 - 1.649)	0.857	0.857
TC/AA	11 (5.0)	41 (10.6)	1.758 (0.803 - 3.847)	0.158	0.316
CC/GG	10 (4.6)	6 (1.5)	0.282 (0.094 - 0.852)	0.025	0.100
CC/GA	10 (4.6)	13 (3.4)	0.616 (0.246 - 1.541)	0.300	0.400
CC/AA	3 (1.4)	4 (1.0)	0.610 (0.126 - 2.961)	0.540	0.540
GP6 rs1671153 /rs1654419					
TT/GG	73 (33.3)	142 (36.6)	1.000 (reference)		
TT/GG	33 (15.1)	59 (15.2)	0.897 (0.537 - 1.500)	0.679	0.811
TT/GG	7 (3.2)	9 (2.3)	0.644 (0.229 - 1.812)	0.405	0.811
TG/GA	53 (24.2)	98 (25.3)	0.948 (0.611 - 1.471)	0.811	0.811
TG/GA	29 (13.2)	51 (13.1)	0.900 (0.525 - 1.543)	0.702	0.811
TG/GA	12 (5.5)	10 (2.6)	0.391 (0.157 - 0.974)	0.044	0.132
GG/AA	6 (2.7)	12 (3.1)	1.066 (0.383 - 2.971)	0.902	0.902
GG/AA	6 (2.7)	5 (1.3)	0.444 (0.131 - 1.511)	0.194	0.291
GG/AA	0 (0.0)	2 (0.5)	-	-	-
GP6 rs1671153 /rs12610286					
TT/AA	53 (24.2)	122 (31.4)	1.000 (reference)		
TT/AA	52 (23.7)	66 (17.0)	0.556 (0.342 - 0.905)	0.018	0.072
TT/AA	8 (3.7)	22 (5.7)	1.224 (0.507 - 2.955)	0.653	0.653
TG/AG	47 (21.5)	87 (22.4)	0.801 (0.495 - 1.296)	0.366	0.488
TG/AG	44 (20.1)	59 (15.2)	0.604 (0.362 - 1.007)	0.053	0.106
TG/AG	3 (1.4)	13 (3.4)	1.680 (0.454 - 6.216)	0.437	0.437
GG/GG	5 (2.3)	6 (1.5)	0.553 (0.160 - 1.910)	0.349	0.437
GG/GG	7 (3.2)	10 (2.6)	0.641 (0.230 - 1.781)	0.393	0.437
GG/GG	0 (0.0)	3 (0.8)	-	-	-
GP6 rs1671153 /rs1654431					
TT/GG	56 (25.6)	85 (21.9)	1.000 (reference)		
TT/GG	51 (23.3)	92 (23.7)	1.170 (0.722 - 1.896)	0.524	0.699
TT/GG	6 (2.7)	33 (8.5)	3.476 (1.360 - 8.885)	0.009	0.036
TG/GA	29 (13.2)	54 (13.9)	1.224 (0.697 - 2.151)	0.482	0.699
TG/GA	50 (22.8)	75 (19.3)	0.970 (0.592 - 1.588)	0.904	0.904
TG/GA	15 (6.8)	30 (7.7)	1.291 (0.636 - 2.622)	0.480	0.640
GG/AA	3 (1.4)	6 (1.5)	1.325 (0.318 - 5.518)	0.700	0.700

GG/AA	7 (3.2)	7 (1.8)	0.658 (0.219 - 1.979)	0.456	0.640
GG/AA	2 (0.9)	6 (1.5)	1.992 (0.387 - 10.242)	0.409	0.640
GP6 rs1654419 /rs12610286					
GG/AA	69 (31.5)	133 (34.3)	1.000 (reference)		
GG/AA	56 (25.6)	90 (23.2)	0.840 (0.539 - 1.310)	0.442	0.553
GG/AA	7 (3.2)	29 (7.5)	2.065 (0.857 - 4.977)	0.106	0.265
GA/AG	31 (14.2)	66 (17.0)	1.079 (0.642 - 1.813)	0.775	0.775
GA/AG	36 (16.4)	41 (10.6)	0.594 (0.348 - 1.014)	0.056	0.265
GA/AG	1 (0.5)	8 (2.1)	4.093 (0.500 - 33.512)	0.189	0.315
AA/GG	5 (2.3)	16 (4.1)	1.551 (0.540 - 4.454)	0.415	0.415
AA/GG	11 (5.0)	4 (1.0)	0.181 (0.055 - 0.595)	0.005	0.015
AA/GG	3 (1.4)	1 (0.3)	0.187 (0.019 - 1.836)	0.150	0.225
GP6 rs1654419 /rs1654431					
GG/GG	58 (26.5)	92 (23.7)	1.000 (reference)		
GG/GG	61 (27.9)	111 (28.6)	1.143 (0.726 - 1.800)	0.563	0.563
GG/GG	13 (5.9)	49 (12.6)	2.203 (1.091 - 4.447)	0.028	0.112
GA/GA	25 (11.4)	48 (12.4)	1.211 (0.675 - 2.173)	0.521	0.563
GA/GA	36 (16.4)	50 (12.9)	0.829 (0.479 - 1.435)	0.503	0.563
GA/GA	7 (3.2)	17 (4.4)	1.535 (0.600 - 3.931)	0.371	0.543
AA/AA	5 (2.3)	5 (1.3)	0.575 (0.157 - 2.114)	0.405	0.543
AA/AA	11 (5.0)	13 (3.4)	0.688 (0.285 - 1.664)	0.407	0.543
AA/AA	3 (1.4)	3 (0.8)	0.645 (0.126 - 3.313)	0.599	0.599
GP6 rs12610286 /rs1654431					
AA/GG	44 (20.1)	78 (20.2)	1.000 (reference)		
AA/GG	53 (24.2)	103 (26.6)	1.101 (0.669 - 1.812)	0.706	0.706
AAG/GG	8 (3.7)	34 (8.8)	2.373 (1.004 - 5.608)	0.049	0.196
AG/GA	40 (18.3)	50 (12.9)	0.715 (0.409 - 1.253)	0.241	0.321
AG/GA	51 (23.3)	62 (16.0)	0.685 (0.406 - 1.156)	0.157	0.314
AG/GA	12 (5.5)	23 (5.9)	1.111 (0.502 - 2.460)	0.795	0.925
GG/AA	4 (1.8)	17 (4.4)	2.466 (0.775 - 7.849)	0.127	0.508
GG/AA	4 (1.8)	8 (2.1)	1.064 (0.296 - 3.823)	0.925	0.925
GG/AA	3 (1.4)	12 (3.1)	2.163 (0.574 - 8.144)	0.254	0.508

Note:AOR = adjusted odds ratio; CI = confidence interval; [a] Fisher's exact test; [b] FDR-adjusted *P* value.

.

Table S4. Haplotype analysis of *GP6* gene polymorphisms in RPL and controls.

Haplotypes	Control (n= 438)	Case (n=776)	OR(95% CI)	*P*[a]	*FDR-P*[b]
***GP6* rs1654410 /*GP6* rs1671153 /*GP6* rs1654419 /*GP6* rs12610286**					
T-T-G-A	109 (24.8)	250 (32.2)	1.000 (reference)		
T-T-G-G	51 (11.7)	90 (11.6)	0.769 (0.510 - 1.160)	0.241	0.301
T-T-A-A	33 (7.5)	61 (7.9)	0.806 (0.499 - 1.302)	0.384	0.384
T-T-A-G	23 (5.3)	24 (3.0)	0.455 (0.246 - 0.841)	0.013	0.032
T-G-G-A	45 (10.3)	50 (6.5)	0.484 (0.305 - 0.769)	0.002	0.010
T-G-G-G	4 (0.9)	32 (4.2)	3.488 (1.204 - 10.110)	0.019	0.032
T-G-A-A	23 (5.2)	35 (4.5)	0.664 (0.374 - 1.176)	0.172	0.287
T-G-A-G	9 (2.0)	0 (0.0)	0.023 (0.001 - 0.399)	<0.0001	0.001
C-T-G-A	76 (17.5)	97 (12.5)	0.557 (0.382 - 0.810)	0.003	0.008
C-T-G-G	12 (2.8)	34 (4.4)	1.235 (0.616 - 2.477)	0.611	0.611
C-T-A-A	6 (1.3)	24 (3.0)	1.744 (0.693 - 4.388)	0.299	0.374
C-T-A-G	10 (2.2)	0 (0.0)	0.021 (0.001 - 0.359)	<0.0001	0.001
C-G-G-A	19 (4.4)	38 (4.9)	0.872 (0.481 - 1.581)	0.646	0.646
C-G-G-G	15 (3.4)	28 (3.6)	0.814 (0.418 - 1.585)	0.601	0.646
C-G-A-A	2 (0.5)	11 (1.5)	2.398 (0.523 - 11.010)	0.359	0.646
C-G-A-G	1 (0.2)	3 (0.4)	1.308 (0.135 - 12.720)	1.000	1.000

Note: ORs and 95% CIs of each allele combinations were calculated with reference to frequencies of all others using Fisher's exact test. *P* value by Fisher's exact test. RPL, recurrent pregnancy loss; OR (odds ratio); CI (confidence interval). [a] Fisher's exact test; [b] FDR-adjusted *P* value.

Table S5. Haplotype analysis of *GP6* gene polymorphisms in RPL and controls.

Haplotypes	Control (n= 438)	Case (n=776)	OR (95% CI)	*P*[a]	*FDR-P*[b]
		GP6 rs1654410 /rs1671153 /rs1654419			
T-T-G	163 (37.2)	338 (43.5)	1.000 (reference)		
T-T-A	54 (12.4)	87 (11.2)	0.777 (0.527 - 1.145)	0.227	0.341
T-G-G	46 (10.5)	83 (10.7)	0.870 (0.580 - 1.306)	0.530	0.530
T-G-A	34 (7.7)	34 (4.3)	0.482 (0.289 - 0.804)	0.006	0.018
C-T-G	84 (19.3)	133 (17.2)	0.764 (0.548 - 1.063)	0.124	0.165
C-T-A	18 (4.1)	21 (2.7)	0.563 (0.292 - 1.085)	0.112	0.165
C-G-G	39 (8.8)	65 (8.4)	0.804 (0.518 - 1.247)	0.361	0.361
C-G-A	0 (0.0)	15 (2.0)	14.970 (0.890 - 252.000)	0.004	0.016
		GP6 rs1654410 /rs1671153			
T-T	216 (49.3)	424 (54.7)	1.000 (reference)		
T-G	81 (18.6)	117 (15.1)	0.736 (0.530 - 1.021)	0.074	0.116
C-T	104 (23.8)	155 (20.0)	0.759 (0.564 - 1.023)	0.077	0.116
C-G	37 (8.4)	80 (10.3)	1.101 (0.722 - 1.681)	0.749	0.749

Note: ORs and 95% CIs of each allele combinations were calculated with reference to frequencies of all others using Fisher's exact test. *P* value by Fisher's exact test. RPL, recurrent pregnancy loss; OR = odds ratio; CI = confidence interval. [a] Fisher's exact test; [b] FDR-adjusted *P* value.

Table S6. Differences of various clinical parameters according to *GP6* gene polymorphisms in RPL control.

**Genotype**		**PLT (10^3/ul)**		**PT (sec)**		**aPTT (sec)**		**BMI (kg/m2)**	**BUN (mg/dL)**	**Creatinine (mg/dl)**	**Uric acid (mg/dL)**	**Total cholesterol (mg/dl)**		**Homocysteine (µmol/L)**		**FSH (mIU/mL)**		**LH (mIU/mL)**		**E2 (pg/mL)**	**PAI-1 (ng/ml)**
	N	Mean ± SD	N	Mean ± SD	N	Mean ± SD	N	Mean ± SD	Mean ± SD	Mean ± SD	Mean ± SD	Mean ± SD	N	Mean ± SD	N	Mean ± SD	N	Mean ± SD	N	Mean ± SD	Mean ± SD
***GP6* rs1654410T>C**																					
TT	70	246.46 ± 64.87	23	0.83 ± 0.08	23	32.94 ± 3.39	38	21.80 ± 3.16	NA	NA	NA	NA	70	36.20 ± 3.72	46	8.55 ± 3.43	45	3.44 ± 2.34	47	27.49 ± 14.13	NA
TC	85	240.88 ± 58.43	21	0.85 ± 0.09	21	32.65 ± 2.94	41	21.60 ± 3.81	NA	NA	NA	NA	85	36.28 ± 4.29	63	7.75 ± 2.10	62	3.36 ± 1.49	63	24.63 ± 15.58	NA
CC	13	226.85 ± 69.27	8	0.89 ± 0.13	8	34.44 ± 2.42	15	21.31 ± 3.27	NA	NA	NA	NA	13	32.95 ± 4.27	2	10.70 ± 5.66	2	2.80 ± 0.42	2	28.85 ± 17.61	NA
P		0.560		0.398		0.374		0.897						0.021		0.144		0.887		0.591	
***GP6* rs1671153 T>G**																					
TT	89	250.39 ± 63.73	31	0.84 ± 0.09	31	32.69 ± 2.94	46	21.48 ± 3.21	NA	NA	NA	NA	89	36.37 ± 3.74	57	8.21 ± 2.60	55	3.48 ± 1.94	58	25.01 ± 13.64	NA
TG	69	230.78 ± 57.04	18	0.86 ± 0.09	18	33.79 ± 3.46	42	21.86 ± 3.76	NA	NA	NA	NA	69	35.64 ± 4.51	48	8.11 ± 3.15	48	3.39 ± 1.86	48	26.56 ± 15.16	NA
GG	10	246.70 ± 71.01	3	0.89 ± 0.17	3	32.33 ± 1.91	6	21.29 ± 3.28	NA	NA	NA	NA	10	35.05 ± 4.81	6	7.58 ± 2.04	6	2.42 ± 1.02	6	29.40 ± 25.44	NA
P		0.138		0.527		0.450		0.851						0.412		0.873		0.420		0.733	
***GP6* rs1654419 G>A**																					
GG	92	231.63 ± 57.91	31	0.85 ± 0.10	31	32.68 ± 2.93	53	21.50 ± 3.12	NA	NA	NA	NA	92	35.79 ± 4.36	59	7.75 ± 2.16	58	3.22 ± 1.86	60	25.12 ± 13.03	NA
GA	58	256.62 ± 63.68	14	0.84 ± 0.09	14	33.70 ± 3.32	32	21.53 ± 4.07	NA	NA	NA	NA	58	36.35 ± 3.80	41	8.26 ± 2.72	40	3.57 ± 1.94	41	29.03 ± 17.11	NA
AA	18	249.00 ± 68.49	7	0.84 ± 0.06	7	33.41 ± 3.51	9	22.75 ± 2.97	NA	NA	NA	NA	18	35.84 ± 4.13	11	9.71 ± 5.14	11	3.55 ± 1.73	11	18.56 ± 14.20	NA
P		0.047		0.899		0.565		0.596						0.711		0.390†		0.645		0.099	
***GP6* rs12610286 A>G**																					
AA	96	249.97 ± 62.89	21	0.86 ± 0.11	21	32.52 ± 3.10	42	21.70 ± 3.05	NA	NA	NA	NA	96	36.48 ± 4.01	72	8.18 ± 3.07	70	3.74 ± 2.13	73	25.08 ± 14.24	NA
AG	61	227.07 ± 58.85	31	0.83 ± 0.08	31	33.41 ± 3.07	52	21.58 ± 3.75	NA	NA	NA	NA	61	34.89 ± 4.36	28	7.83 ± 1.97	28	2.84 ± 1.03	28	28.15 ± 17.41	NA
GG	11	257.00 ± 57.83	NA	NA	NA	NA	NA	NA	NA	NA	NA	NA	11	37.80 ± 2.34	11	8.59 ± 2.95	11	2.46 ± 0.94	11	25.71 ± 13.47	NA
P		0.054		0.252		0.311		0.870						0.026†		0.728		0.049†		0.655	
***GP6* rs1654431 G>A**																					
GG	57	244.02 ± 59.45	25	0.85 ± 0.09	25	32.64 ± 3.15	44	21.82 ± 3.08	NA	NA	NA	NA	57	36.14 ± 3.73	31	7.70 ± 1.90	29	3.01 ± 1.97	31	28.07 ± 14.50	NA
GA	83	239.20 ± 64.89	20	0.83 ± 0.08	20	33.78 ± 3.36	37	20.71 ± 2.56	NA	NA	NA	NA	83	36.06 ± 4.23	61	8.31 ± 3.34	61	3.44 ± 1.87	62	25.51 ± 14.86	NA
AA	28	246.89 ± 59.15	7	0.89 ± 0.12	7	32.44 ± 1.58	13	23.64 ± 5.57	NA	NA	NA	NA	28	35.49 ± 4.69	19	8.30 ± 2.10	19	3.77 ± 1.70	19	23.68 ± 16.24	NA
P		0.818		0.415		0.407		0.163†						0.778		0.597		0.366		0.578	

Note: SD, standard deviation; aPTT, Activated Partial Thromboplastin Time; PT, prothrombin time; WBC, white blood cell; Hgb, hemoglobin; PLT, platelet count; BUN, blood urea nitrogen; NA = not applicable; [a] Calculated using ANOVA [b] Calculated using the Kruskal-Wallis test.

Table S7. Differences of various clinical parameters according to *GP6* gene polymorphisms in RPL patients.

Genotype		PLT (10^3/ul)		PT (sec)		aPTT (sec)		BMI (kg/m2)		BUN (mg/dL)		Creatinine (mg/dl)		Uric acid (mg/dL)		Total cholesterol (mg/dl)		Homocysteine (μmol/L)		FSH (mIU/mL)		LH (mIU/mL)		E2 (pg/mL)		PAI-1 (ng/ml)
	N	Mean ± SD	N	Mean ± SD	N	Mean ± SD	N	Mean ± SD	N	Mean ± SD	N	Mean ± SD	N	Mean ± SD	N	Mean ± SD	N	Mean ± SD	N	Mean ± SD	N	Mean ± SD	N	Mean ± SD	N	Mean ± SD
***GP6* rs1654410T>C**																										
TT	101	250.01 ± 61.48	99	0.99 ± 0.10	99	32.87 ± 4.00	153	19.72 ± 7.58	98	9.77 ± 2.75	97	0.73 ± 0.12	84	3.83 ± 0.75	85	181.24 ± 46.96	101	36.77 ± 3.40	88	8.41 ± 12.72	88	6.20 ± 7.75	79	34.92 ± 30.16	61	10.84 ± 5.73
TC	92	262.90 ± 58.26	97	0.97 ± 0.09	97	31.68 ± 4.65	135	19.98 ± 5.92	88	10.20 ± 2.74	88	0.73 ± 0.13	80	3.80 ± 0.96	83	190.28 ± 49.73	91	37.90 ± 3.29	99	6.06 ± 2.62	100	4.86 ± 2.99	82	37.32 ± 29.74	61	10.32 ± 5.86
CC	12	243.83 ± 41.46	14	0.97 ± 0.11	14	31.67 ± 3.86	20	18.79 ± 6.88	12	10.23 ± 3.19	12	0.68 ± 0.14	12	3.60 ± 0.48	12	216.17 ± 56.64	12	37.30 ± 3.15	10	14.11 ± 26.34	10	21.52 ± 47.31	7	25.69 ± 15.06	6	9.46 ± 4.86
P		0.251		0.309		0.135		0.763		0.545		0.384		0.672		0.058		0.067		0.245†		0.228†		0.577		0.793
***GP6* rs1671153 T>G**																										
TT	124	253.57 ± 63.06	123	0.98 ± 0.10	123	31.97 ± 4.54	170	19.68 ± 6.25	122	10.04 ± 2.74	122	0.72 ± 0.12	109	3.82 ± 0.79	111	188.14 ± 50.33	123	36.81 ± 3.40	110	8.34 ± 13.59	111	6.00 ± 7.88	99	35.74 ± 33.90	77	11.01 ± 6.04
TG	72	259.26 ± 54.04	77	0.99 ± 0.10	77	32.81 ± 4.07	123	19.70 ± 7.91	68	9.88 ± 2.71	67	0.72 ± 0.13	61	3.78 ± 0.95	63	189.67 ± 48.47	72	38.18 ± 3.02	80	6.61 ± 4.09	80	6.73 ± 16.60	65	36.06 ± 22.32	46	9.71 ± 5.37
GG	9	250.56 ± 46.52	10	0.94 ± 0.08	10	31.11 ± 3.18	15	21.43 ± 2.19	8	10.05 ± 3.88	8	0.78 ± 0.15	6	3.75 ± 0.33	6	160.00 ± 39.98	9	37.09 ± 4.41	7	4.95 ± 2.86	7	5.98 ± 5.09	4	29.03 ± 8.27	5	10.66 ± 3.19
P		0.786		0.263		0.287		0.630		0.927		0.477		0.954		0.371		0.022		0.432		0.917		0.899		0.480
***GP6* rs1654419 G>A**																										
GG	134	252.22 ± 56.95	136	0.98 ± 0.11	136	32.20 ± 4.17	202	19.84 ± 7.15	128	9.87 ± 2.81	128	0.72 ± 0.12	112	3.77 ± 0.81	116	185.84 ± 47.75	134	37.30 ± 3.33	125	7.91 ± 12.85	125	5.82 ± 7.81	105	35.83 ± 32.60	76	9.57 ± 5.47
GA	61	255.28 ± 57.83	62	0.98 ± 0.09	62	32.17 ± 4.49	89	19.76 ± 6.41	59	10.24 ± 2.69	58	0.76 ± 0.12	55	3.84 ± 0.88	55	187.15 ± 50.40	60	37.28 ± 3.54	64	6.99 ± 4.20	64	7.45 ± 18.27	57	35.35 ± 24.21	46	11.96 ± 5.96
AA	10	299.40 ± 83.44	12	0.97 ± 0.08	12	33.02 ± 5.43	17	19.12 ± 5.28	11	10.01 ± 2.80	11	0.65 ± 0.09	9	3.99 ± 0.94	9	215.78 ± 61.46	10	37.66 ± 3.18	8	5.58 ± 3.00	9	4.73 ± 2.76	6	36.98 ± 17.68	6	11.71 ± 5.40
P		0.051		0.919		0.813		0.916		0.693		0.012		0.679		0.216		0.944		0.740		0.630		0.990		0.072
***GP6* rs12610286 A>G**																										
AA	100	256.26 ± 58.18	107	1.00 ± 0.09	107	32.32 ± 4.51	167	19.32 ± 7.66	102	10.00 ± 2.82	102	0.74 ± 0.13	92	3.87 ± 0.90	96	185.69 ± 47.13	99	37.17 ± 3.36	105	8.02 ± 11.32	105	7.05 ± 15.64	99	37.22 ± 34.95	75	11.75 ± 6.01
AG	88	257.25 ± 55.85	86	0.98 ± 0.11	86	32.48 ± 4.26	113	20.91 ± 4.96	81	10.03 ± 2.73	80	0.71 ± 0.11	72	3.70 ± 0.75	72	187.78 ± 50.81	88	37.66 ± 3.32	79	6.77 ± 10.16	79	5.48 ± 6.33	55	32.34 ± 19.44	44	7.89 ± 4.34
GG	17	241.18 ± 81.10	17	0.95 ± 0.09	17	30.49 ± 3.02	28	17.87 ± 7.59	15	9.67 ± 2.84	15	0.69 ± 0.16	12	3.87 ± 0.85	12	203.83 ± 59.75	17	36.27 ± 3.58	13	7.99 ± 4.66	14	5.30 ± 2.85	14	38.28 ± 17.33	9	13.26 ± 4.88

P		0.583		0.109		0.214		0.09†		0.895		0.297		0.451		0.490		0.253		0.718		0.653		0.583		0.0003†
GP6 **rs1654431 G>A**																										
GG	75	259.01 ± 63.57	75	0.99 ± 0.10	75	32.24 ± 4.10	131	20.36 ± 6.26	73	9.42 ± 2.34	72	0.70 ± 0.12	62	3.74 ± 0.73	65	196.17 ± 53.48	75	36.91 ± 2.96	59	6.88 ± 11.20	59	5.90 ± 7.03	50	35.38 ± 35.67	46	9.68 ± 5.73
GA	105	258.77 ± 57.26	108	0.98 ± 0.10	108	32.47 ± 4.54	144	20.07 ± 6.14	102	10.20 ± 2.93	102	0.73 ± 0.12	96	3.84 ± 0.88	98	181.94 ± 48.19	105	37.56 ± 3.50	104	6.53 ± 3.66	104	4.78 ± 2.66	88	32.85 ± 21.85	64	10.88 ± 5.86
AA	25	230.68 ± 49.18	27	0.99 ± 0.11	27	31.32 ± 4.07	33	16.12 ± 10.27	23	10.83 ± 3.04	23	0.77 ± 0.12	18	3.82 ± 0.98	17	188.88 ± 36.08	24	37.46 ± 3.95	34	11.66 ± 19.34	35	11.49 ± 26.57	30	44.63 ± 36.17	18	11.48 ± 5.26
P		0.083		0.637		0.466		0.529†		0.055		0.042		0.772		0.197		0.431		0.001†		0.434†		0.167		0.421

Note: SD, standard deviation; aPTT, Activated Partial Thromboplastin Time; PT, prothrombin time; WBC, white blood cell; Hgb, hemoglobin; PLT, platelet count; BUN, blood urea nitrogen; NA = not applicable; [a] Calculated using ANOVA [b] Calculated using the Kruskal-Wallis test.

Table S8. Clinical variables in RSA patients and controls stratified by *GP6* polymorphisms status by ANOVA.

Characteristics	PLT (10^3/ul)		PT (sec)		aPTT (sec)		BMI (kg/m2)		Creatinine (mg/dl)		Total cholesterol (mg/dl)		Homocysteine (μmol/L)		FSH (mIU/mL)		PAI-1 (ng/ml)	
	N	Mean ± SD	N	Mean ± SD	N	Mean ± SD	N	Mean ± SD	N	Mean ± SD	N	Mean ± SD	N	Mean ± SD	N	Mean ± SD	N	Mean ± SD
***GP6* rs1654410T>C**																		
TT	171	248.56 ± 62.72	122	0.96 ± 0.12	122	32.88 ± 3.88	191	20.13 ± 6.97	97	0.73 ± 0.12	85	181.24 ± 46.96	171	36.54 ± 3.54	134	8.46 ± 10.48	61	10.84 ± 5.73
TC	177	252.33 ± 59.21	118	0.95 ± 0.11	118	31.85 ± 4.40	176	20.36 ± 5.53	88	0.73 ± 0.13	83	190.28 ± 49.73	176	37.12 ± 3.88	162	6.72 ± 2.56	61	10.32 ± 5.86
CC	25	235.00 ± 57.12	22	0.94 ± 0.12	22	32.68 ± 3.61	35	19.87 ± 5.70	12	0.68 ± 0.14	12	216.17 ± 56.64	25	35.04 ± 4.31	12	13.55 ± 23.92	6	9.46 ± 4.86
P		0.398		0.533		0.143		0.893		0.384		0.058		0.026		0.232†		0.793
C allele	348	250.47 ± 60.91	240	0.96 ± 0.11	240	32.38 ± 4.17	367	20.24 ± 6.31	185	0.73 ± 0.12	168	185.70 ± 48.41	347	36.83 ± 3.72	296	7.51 ± 7.34	122	10.58 ± 5.78
T allele	25	235.00 ± 57.12	22	0.94 ± 0.12	22	32.68 ± 3.61	35	19.87 ± 5.70	12	0.68 ± 0.14	12	216.17 ± 56.64	25	35.04 ± 4.31	12	13.55 ± 23.92	6	9.46 ± 4.86
P		0.219		0.513		0.744		0.739		0.168		0.039		0.022		0.239†		0.642
***GP6* rs1654419 G>A**																		
GG	226	243.84 ± 58.11		0.96 ± 0.12	167	32.29 ± 3.97	255	20.18 ± 6.55	128	0.72 ± 0.12	116	185.84 ± 47.75	226	36.68 ± 3.84	184	7.86 ± 10.64	76	9.57 ± 5.47
GA	119	255.93 ± 60.49		0.96 ± 0.10	76	32.45 ± 4.32	121	20.23 ± 5.92	58	0.76 ± 0.12	55	187.15 ± 50.40	118	36.83 ± 3.68	105	7.49 ± 3.73	46	11.96 ± 5.96
AA	28	267.00 ± 76.68		0.92 ± 0.10	19	33.17 ± 4.71	26	20.37 ± 4.87	11	0.65 ± 0.09	9	215.78 ± 61.46	28	36.49 ± 3.86	19	7.97 ± 4.75	6	11.71 ± 5.40
P		0.060		0.404		0.676		0.989		0.012		0.216		0.900		0.931		0.072
G allele	345	267.00 ± 76.68	243	0.92 ± 0.10	243	33.17 ± 4.71	376	20.37 ± 4.87	186	0.65 ± 0.09	171	215.78 ± 61.46	344	36.49 ± 3.86	289	7.97 ± 4.75	122	11.71 ± 5.40
A allele	28	248.01 ± 59.14	19	0.96 ± 0.11	19	32.34 ± 4.07	26	20.20 ± 6.35	11	0.73 ± 0.12	9	186.26 ± 48.47	28	36.73 ± 3.78	19	7.73 ± 8.78	6	10.47 ± 5.75
P		0.112		0.179		0.401		0.889		0.033		0.081		0.749		0.904		0.608
***GP6* rs12610286 A>G**																		
AA	196	253.18 ± 60.46	128	0.97 ± 0.10	128	32.36 ± 4.30	209	19.80 ± 7.05	102	0.74 ± 0.13	96	185.69 ± 47.13	195	36.83 ± 3.70	177	8.09 ± 8.91	75	11.75 ± 6.01
AG	149	244.90 ± 58.82	117	0.94 ± 0.12	117	32.73 ± 3.99	165	21.12 ± 4.61	80	0.71 ± 0.11	72	187.78 ± 50.81	149	36.53 ± 4.01	107	7.05 ± 8.79	44	7.89 ± 4.34
GG	28	247.39 ± 72.10	17	0.95 ± 0.09	17	30.49 ± 3.02	28	17.87 ± 7.59	15	0.69 ± 0.16	12	203.83 ± 59.75	28	36.87 ± 3.19	24	8.26 ± 3.90	9	13.26 ± 4.88
P		0.448		0.045		0.110		0.183†		0.297		0.490		0.747		0.584		0.0003†
A allele	345	249.60 ± 59.81	245	0.96 ± 0.11	245	32.53 ± 4.15	374	20.38 ± 6.12	182	0.73 ± 0.12	168	186.58 ± 48.60	344	36.70 ± 3.83	284	7.70 ± 8.86	119	10.32 ± 5.75
G allele	28	247.39 ± 72.10	17	0.95 ± 0.09	17	30.49 ± 3.02	28	17.87 ± 7.59	15	0.69 ± 0.16	12	203.83 ± 59.75	28	36.87 ± 3.19	24	8.26 ± 3.90	9	13.26 ± 4.88
P		0.853		0.688		0.047		0.040		0.338		0.244		0.818		0.756		0.138
***GP6* rs1654431 G>A**																		
GG	132	252.54 ± 62.04	100	0.95 ± 0.11	100	32.34 ± 3.87	175	20.73 ± 5.66	72	0.70 ± 0.12	65	196.17 ± 53.48	132	36.58 ± 3.32	90	7.16 ± 9.11	46	9.68 ± 5.73
GA	188	250.13 ± 61.35	128	0.95 ± 0.11	128	32.68 ± 4.39	181	20.20 ± 5.59	102	0.73 ± 0.12	98	181.94 ± 48.19	188	36.90 ± 3.90	165	7.18 ± 3.64	64	10.88 ± 5.86
AA	53	239.25 ± 54.77	34	0.97 ± 0.12	34	31.55 ± 3.70	46	18.25 ± 9.74	23	0.77 ± 0.12	17	188.88 ± 36.08	52	36.40 ± 4.44	53	10.46 ± 15.54	18	11.48 ± 5.26
P		0.395		0.750		0.360		0.994†		0.042		0.197		0.619		0.001†		0.421
G allele	320	239.25 ± 54.77	228	0.97 ± 0.12	228	31.55 ± 3.70	356	18.25 ± 9.74	174	0.77 ± 0.12	163	188.88 ± 36.08	320	36.40 ± 4.44	255	10.46 ± 15.54	110	11.48 ± 5.26
A allele	53	251.12 ± 61.55	34	0.95 ± 0.11	34	32.53 ± 4.17	46	20.46 ± 5.62	23	0.72 ± 0.12	17	187.61 ± 50.69	52	36.76 ± 3.67	53	7.18 ± 6.14	18	10.38 ± 5.81
P		0.187		0.448		0.196		0.921†		0.054		0.920		0.521		0.002		0.450

ANOVA, analysis of variance; SD, standard deviation. *Calculated using ANOVA. †Calculated using the Kruskal-Wallis test.

Table S9. Frequencies of GP6 polymorphisms in world-wide populations.

**Population**		**GP6 rs1654410 C>T**			Allele frequencies	
	**n**	**TT(%)**	TC(%)	CC(%)	T	C
European	48	8 (16.7)	28 (58.3)	12 (25.0)	0.458	0.542
Asian (Japanese)	90	16 (17.8)	52 (57.8)	22 (24.4)	0.467	0.533
Sub-Saharan African	120	68 (56.7)	34 (28.3)	18 (15.0)	0.708	0.292
Asian (Japanese)	172	26(15.1)	100(58.1)	46 (26.7)	0.442	0.558
Asian (Chinese)	90	12(13.3)	42(46.7)	36(40.0)	0.367	0.633
Asian (Korean)	219	88(40.2)	108(49.3)	23(10.5)	0.648	0.352
Population		GP6 rs1671153T>G			Allele frequencies	
	n	TT(%)	TG(%)	GG(%)	T	G
European	226	150 (66.3)	60 (26.5)	16 (7.0)	0.787	0.212
Asian (Japanese)	172	92 (35.3)	68 (58.8)	12 (5.3)	0.732	0.267
Sub-Saharan African	226	40 (17.6)	118 (52.2)	68 (30.0)	0.438	0.561
African	44	22 (9.7)	12(5.3)	10(4.4)	0.636	0.363
Asian (Chinese)	86	64(74.4)	22(25.5)		0.872	0.127
African	2		2(100)		0.500	0.500
European	46	36(78.2)	6(13.0)	4(8.6)	0.847	0.152
Asian (Chinese)	82	58(70.7)		24(29.2)	0.853	0.146
Asian (Korean)	219	107(48.9)	98(44.7)	14(6.4)	0.712	0.288
Population		GP6 rs1654419 G>A			Allele frequencies	
	n	GG(%)	GA(%)	AA(%)	G	A
European	224	22(9.8)	56(25.0)	146 (65.2)	0.223	0.223
Asian (Japanese)	172	12 (7.0)	68 (39.5)	92 (53.5)	0.267	0.267
Sub-Saharan African	120	46 (20.4)	122 (54.0)	58 (25.7)	0.473	0.473
Asian (Korean)	219	122(55.7)	77(35.2)	20(9.1)	0.733	0.267
Population		GP6 rs12610286 A>G			Allele frequencies	
	n	AA(%)	AG(%)	GG(%)	A	G
European	226	134 (59.3)	82 (36.3)	10 (4.4)	0.774	0.226
Asian (Japanese)	172	42(24.4)	100 (58.1)	30 (17.4)	0.535	0.465
Sub-Saharan African	226	98 (43.4)	110 (48.7)	18 (8.0)	0.677	0.323
Asian (Chinese)	82	12(14.6)	34(41.5)	36(43.9)	0.354	0.646
Asian (Korean)	219	118(53.9)	90(41.10	11(5.0)	0.744	0.256
Population		GP6 6 rs1654431 G>A			Allele frequencies	
	n	GG(%)	GA(%)	AA(%)	G	A
European	226	42 (59.3)	118(36.3)	66 (4.4)	0.447	0.553
Asian (Japanese)	170	46(27.1)	92 (54.1)	32 (18.8)	0.541	0.459
Sub-Saharan African	226	18 (8.0)	102 (45.1)	106 (46.9)	0.305	0.695
Asian (Chinese)	86	28(32.6)	42(48.8)	16(18.6)	0.570	0.430
Asian (Korean)	219	79(36.1)	104(47.5)	36(16.4)	0.598	0.402
